# Automated recognition of epilepsy from EEG signals using a combining space–time algorithm of CNN-LSTM

**DOI:** 10.1038/s41598-023-41537-z

**Published:** 2023-09-08

**Authors:** Xiashuang Wang, Yinglei Wang, Dunwei Liu, Ying Wang, Zhengjun Wang

**Affiliations:** The Second Academy of China Aerospace Science and Industry Corporation (CASIC), 50 Yongding Road, Haidian District, Beijing, China

**Keywords:** Computational neuroscience, Network models, Computational biology and bioinformatics, Neuroscience, Diseases

## Abstract

Intelligent recognition methods for classifying non-stationary and non-invasive epileptic diagnoses are essential tools in neurological research. Electroencephalogram (EEG) signals exhibit better temporal characteristics in the detection of epilepsy compared to radiation medical images like computed tomography (CT) and magnetic resonance imaging (MRI), as they provide real-time insights into the disease’ condition. While classical machine learning methods have been used for epilepsy EEG classification, they still often require manual parameter adjustments. Previous studies primarily focused on binary epilepsy recognition (epilepsy vs. healthy subjects) rather than as ternary status recognition (continuous epilepsy vs. intermittent epilepsy vs. healthy subjects). In this study, we propose a novel deep learning method that combines a convolution neural network (CNN) with a long short-term memory (LSTM) network for multi-class classification including both binary and ternary tasks, using a publicly available benchmark database on epilepsy EEGs. The hybrid CNN-LSTM automatically acquires knowledge without the need for extra pre-processing or manual intervention. Besides, the joint network method benefits from memory function and stronger feature extraction ability. Our proposed hybrid CNN-LSTM achieves state-of-the-art performance in ternary classification, outperforming classical machine learning and the latest deep learning models. For the three-class classification, in the method achieves an accuracy, specificity, sensitivity, and ROC of 98%, 97.4, 98.3% and 96.8%, respectively. In binary classification, the method achieves better results, with ACC of 100%, 100%, and 99.8%, respectively. Our dual stream spatiotemporal hybrid network demonstrates superior performance compared to other methods. Notably, it eliminates the need for manual operations, making it more efficient for doctors to diagnose during the clinical process and alleviating the workload of neurologists.

## Introduction

Epilepsy is a chronic characterized by sudden abnormal brain neuron discharge, leading to transient brain dysfunction^1^. It affects approximately 50 million people worldwide, including adults, infants, and young children. Epileptic individuals present various symptoms, including generalized convulsions, unconsciousness, and urinary incontinence. The condition often occurs unexpectedly and without warning, potentially causing irreversible brain damage and life-threatening situations. Therefore, early diagnosis and precautions for epilepsy are of utmost importance. Medical imaging techniques like CT and MRI can detect epilepsy. Although CT and MRI can detect epilepsy by identifying lesions and providing spatial information. However, they lack temporal data and cannot detect persistent seizure. Moreover, MRI is expensive and time-consuming, limiting limiting its application, particulary for certain groups that are required to lie down quietly during testing. To address these limitations and ensure timely epilepsy diagnosis, electroencephalogram (EEG) signals are utilized for epilepsy recognition. EEG examinations are highly accurate in positioning^2^, and they effectively address the challenges of persistent epilepsy EEG examination. Accurate decoding of epileptic EEG is vital for auxiliary medical epilepsy diagnosis reducing the risk of seizures^3^, and supporting neurologists in busy neurological wards.

Previous studies, involved extracting features from complex EEG using conventional signal processing and then adopting supervised learning paradigms for epileptic feature recognition. Since epilepsy encompasses various types, labeling EEG and training classification models based on known types or degrees of epilepsy is essential^4^. Two key factors influence the accuracy of epilepsy recognition: the feature extractor and the feature classifier. Most EEG recognition is based on binary classification distinguishing epileptic individuals from healthy subjects. Multi-classification, such as distinguishing between continuous epilepsy, intermittent epilepsy, and healthy subjects, is relatively rare. In the study of the binary classification of epileptic EEG, several methods have been proposed, including the Welch spectral mining technique for extracting the epilepsy features by calculating the time domain information of the EEG signals during epileptic seizures^5^. In addition, an improved Fourier method for spectral analysis has gained popularity in analyzing the frequency information of EEG. Moreover, Polat et al. combined a fast Fourier transform model with a decision trees model for seizure feature extraction^6^. Additionally, Bauquier et al. presented a seminal article proposing essential research for automated EEG-based epilepsy recognition by applying spike-and-wave complex recognition algorithm^7^. Flexible wavelet transforms, and the fractal dimension of the time–frequency models have been used for seizure segment analysis in long-term EEG recognition^8,9^. Ashokkumar S.R. et al. also provided a wavelet filter bank to satisfy the regularity criteria. The performances of the proposed work using the Bonn EEG set obtain high result and ensure validation concerning compatibility and robustness^10^.

Emami Ali et al. hypothesized that epileptologists could recognize seizures by visually analyzing EEG plots^11^. Similarly, Liu et al. also used a method of visual inspection features to address the recognition problem^12^. Support vector machines (SVM) have been widely used for classifying the epilepsy subjects’ EEG as proposed by Wang et al.^13^. Brabanter et al. improved the SVM and proposed a least squares support vector machine (LS-SVM) algorithm for classifying two levels of seizure and non-seizure EEG from the small sample epilepsy datasets of Bonn University, Germany. They achieved better results, that is, 98.0–99.5% and 99.5–100% accuracy using a radial basis function (RBF) kernel and a Morlet kernel, respectively^14^. However, selecting of a suitable classifier remains a significant challenge, and various strategies have been developed for seizure recognition, including K-nearest neighbors (KNN)^15^, empirical mode decomposition (EMD)^16^, random forests (RF)^17^, and reinforcement learning^18^. Researchers have often found suitable classifiers for medical diagnosis systems through trial and error, achieving high identification accuracy of epilepsy patterns, ranging from 93.0 to 99.6%. Despite the success of binary identification methods, they may not be practical for real-world applications, necessitating the introduction of multi-class classification approaches for epilepsy recognition. Shafiul Alam et al. proposed a method utilizing higher order statistical moments of EEG, calculated using the EMD method, for detecting seizure and epilepsy. They achieved 80% accuracy using an artificial neural network (ANN) as the classifier with these moments. More recently, Ilakiyaselvan N et al. studied another ternary epilepsy recognition problem, distinguishing continuous ictal epilepsy patients, intermittent epilepsy patients, and healthy subjects by using areconstructed phase space images and a convolution neural network (CNN), resulting in an accuracy of 93.9% is achieved. The rationale of deep learning lies in building and simulating the neural networks that emulate the human brain’s mechanisms for analytical learning. This approach mimics the human mind’s ability to interpret various types of data, such as images, videos and text^19^. MohanBabu G. et al. introduced an optimized deep learning network (ODLN) methodology that significantly improves in the performance rate of seizure prediction when compared with independent CNN algorithm^20^. Acharya U R. et al. have designed a 13-layer deep CNN to classify the three EEG classes for epilepsy patients^21^. However, EEG signals encompass both temporal and spatial features, and the independent CNN algorithm overlooks the temporal continuity of EEG data. Since the existing CNN is better in processing two-dimensional image recognition, they may not be ideally suited for handling the multidimensional signals present in EEG.

Our work proposes a novel approach for classifying epileptic EEG signals by combining CNN with LSTM network within a bidirectional recurrent neural network (BRNN). The main idea of the CNN-LSTM combined algorithm is to use convolution layers as the initial network to receive epileptic EEG signals as input. The algorithm improves by aggregating the output of multi-layer convolutions into a smaller size. Next, we connect the Max pooling layer to the dropout layer, followed by the construction of an LSTM layer, which receives the output of the dropout layer down to the LSTM layer. The LSTM layer uses CNN to extract the spatial features of epileptic EEG signals for sorting. This process involves memorizing and forgetting EEG features, effectively capturing temporal characteristics. Finally, we connect the LSTM layer to the fully connected layer, where the output of the LSTM layer serves as the input to the fully connected layer. The hybrid spatio-temporal neural network bundled in this way requires more epochs to learn effectively reducing over-fitting. The advantage of this dual stream spatio-temporal neural network, CNN-LSTM, over independent CNN or LSTM model lies in its stronger learning and memory capabilities. The CNN-LSTM method can “remember” the previous state of the EEG signal, enabling recognition based on current state change trend. Due to its ability to capture more comprehensive information, this method often achieves higher accuracy and effectively addresses issues like the exploding or vanishing gradient problem. Moreover, CNN-LSTM is versatile and suitable for different types, offering a promising new approach for epilepsy identification in the auxiliary medical diagnostic system.

The remainder of this paper is organized as follows. In Sect. “Materials and methods**”**, the auxiliary medical diagnostic system is described, and our novel automatic recognition scheme for epileptic EEG based on dual stream combination algorithm CNN-LSTM is presented. Details about the experimental data are also provided in the Sect. “Experimental data”. The experimental results are analyzed, using deep learning-based to assessment indices, and compared with existing research states in Sect. “Results”. Lastly, the contributed innovation and planned future work are summarized in Sect. “Discussion” and Sect. “Conclusions”, respectively.

## Materials and methods

### Auxiliary medical diagnostic system

**Figure 1:**
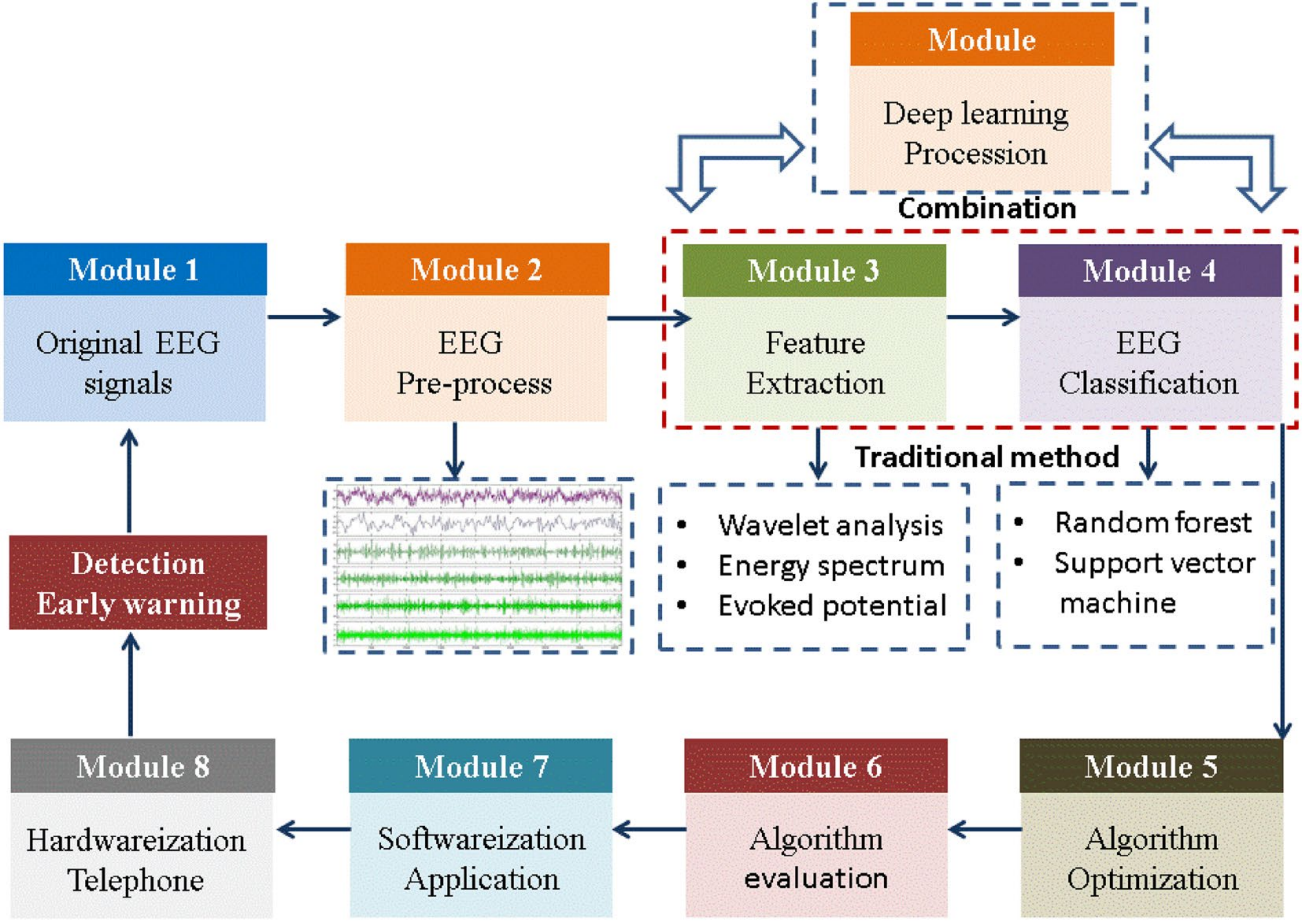
Traditional medical auxiliary system framework for diagnosing epilepsy EEG.

Generally, a traditional epilepsy detection system consists of the following 8 modules is shown in Fig. 1. After obtaining the original epilepsy EEG signals in the ward, which cannot be directly used for epilepsy recognition. Firstly, it is necessary to pre-process the EEG, which is accompanied by a large amount of noise and interference during the collection. The pre-processing module mainly performs filtering, removing bad channels, baseline correction, and re referencing operations on the collected EEG signals. At the same time, independent component analysis is used to remove various types of artifacts from the signals. In the traditional system of epilepsy recognition, it includes two main modules: feature extraction and EEG classification module. We can consider using algorithms such as wavelet transform, power spectrum, and time-dependent potential to extract EEG features. These features are used as inputs for module 4th of EEG classifier. A large number of hyper-parameters are generated during algorithm training, so module 5th is essential for built an optimization that can automatically search the most suitable hyper-parameters. In module 6th, evaluating the effectiveness of classification results requires indicators to measure, such as accuracy (ACC), confusion matrix (CM), precision recall curve (PRC), a receiver operating characteristic (ROC) curve^22,23^, and the area under the curve (AUC)^24^ and recall curve^25^. The above modules are all the part of processing data. The combined algorithms can be developed into software for mobile applications, and then hardware can be used to produce real-time epilepsy detection devices, playing a role in monitoring and early warning. However, deep learning (DL) technologies are used in the medical information processing, which combine the module 3th, module 4th and module 5th. Because of the module of DL can learn the relative features, recognize the features even adjust hyper-parameters adaptively by it-self. DL can better support calculations with GPUs, which is more time-saving and labor-saving.

**Figure 2:**
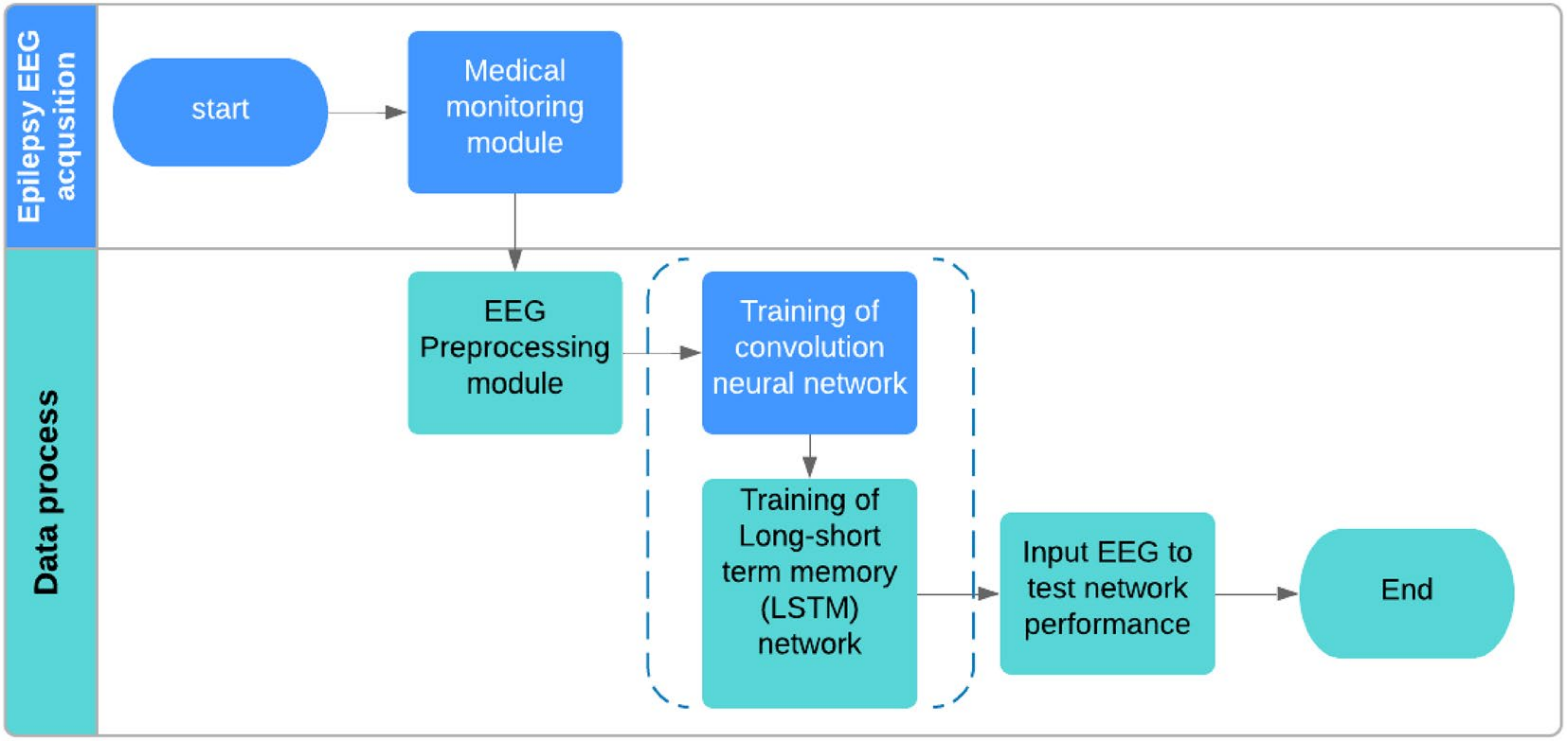
Auxiliary diagnosis for epileptic EEG based on a CNN-LSTM model.

We mainly discuss the four major modules of auxiliary diagnostic systems in this paper. As illustrated in Fig. 2. The first step, involves obtaining raw EEG signals enter the pre-processing module. In the next step, the signals are fed into the automatic extraction and classification module, which combines CNN and LSTM network. Pre-processed data are input into the convolution and pooling layers to extract spatial features. Subsequently, the LSTM is directly connected to the pooling layer to extract the time series information of the EEG data. Finally, the dropout and fully connected layers are used to complete the classification task. Multiple indices are used along with a verification module to evaluate the performance of the proposed scheme in the three seizure status classifications.

### Methodology

#### Pre-processing

The raw EEG data were recorded using a standard 10–20 system with a sampling frequency of 173.61 Hz. To obtain the useful frequency range, band-pass filtering was applied, resulting in a frequency range of 0.5 to 50 Hz^26^. Additionally, the EEG dataset was segmented by 23.6s duration. Then, it was normalized to have zero mean and unit standard deviation, before being fed into the model.

In this work, a GPU was used for calculation. To implement the hybrid CNN-LSTM model, it was necessary to install the Python-based deep learning library Keras on the server and use TensorFlow, Google’s deep learning framework, as the back-end. The model was then trained accordingly. While deep learning network has models possess strong learning capabilities, certain parameters needed to be set based on model’s requirements and empirical experience to optimize the speed and achieve higher ACC.

#### Convolution neural network

**Figure 3:**
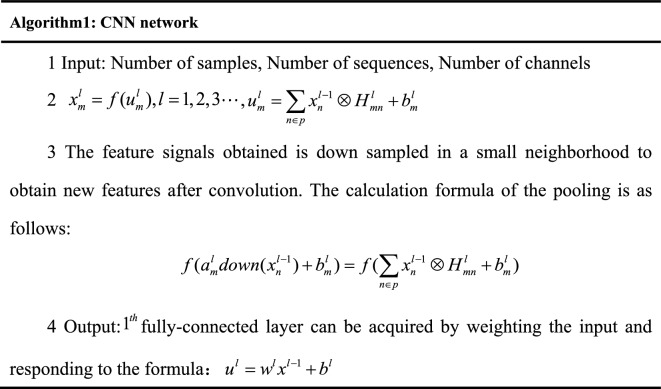
Pseudocode of CNN algorithm. where $u_{m}^{l}$ represent the $m^{th}$ channel activation value of the $1^{th}$ convolutional layer, $u_{m}^{l}$ is obtained by the convolution of the upper layer and bias, $x_{m}^{l}$ is the $m^{th}$ channel output of $1^{th}$ convolution layer, $f( \cdot )$ is the activation function, $p$ is the selected feature sets of input signals, $H_{mn}^{l}$ represents a convolution function, $b_{m}^{l}$ is the bias of $u_{m}^{l}$, $down( \cdot )$ represents the $down$ sample function, $a_{m}^{l}$ is the offset coefficient, $a_{m}^{1}$, $b_{m}^{1}$ are the bias coefficient of the feature, $u^{l}$ is the activation value of the $l^{th}$ fully-connected layer, $w^{l}$ represent the weight of the fully-connected layer, and $b^{l}$ are bias.

The spatial characteristics of EEG data are extracted using the CNN network. The pre-processed EEG are reconstructed into three dimensions, and then input into the CNN network for feature extraction. The architecture of the CNN can be expressed described using algorithm implementation shown in Fig. 3.

#### Long-short term memory network

**Figure 4:**
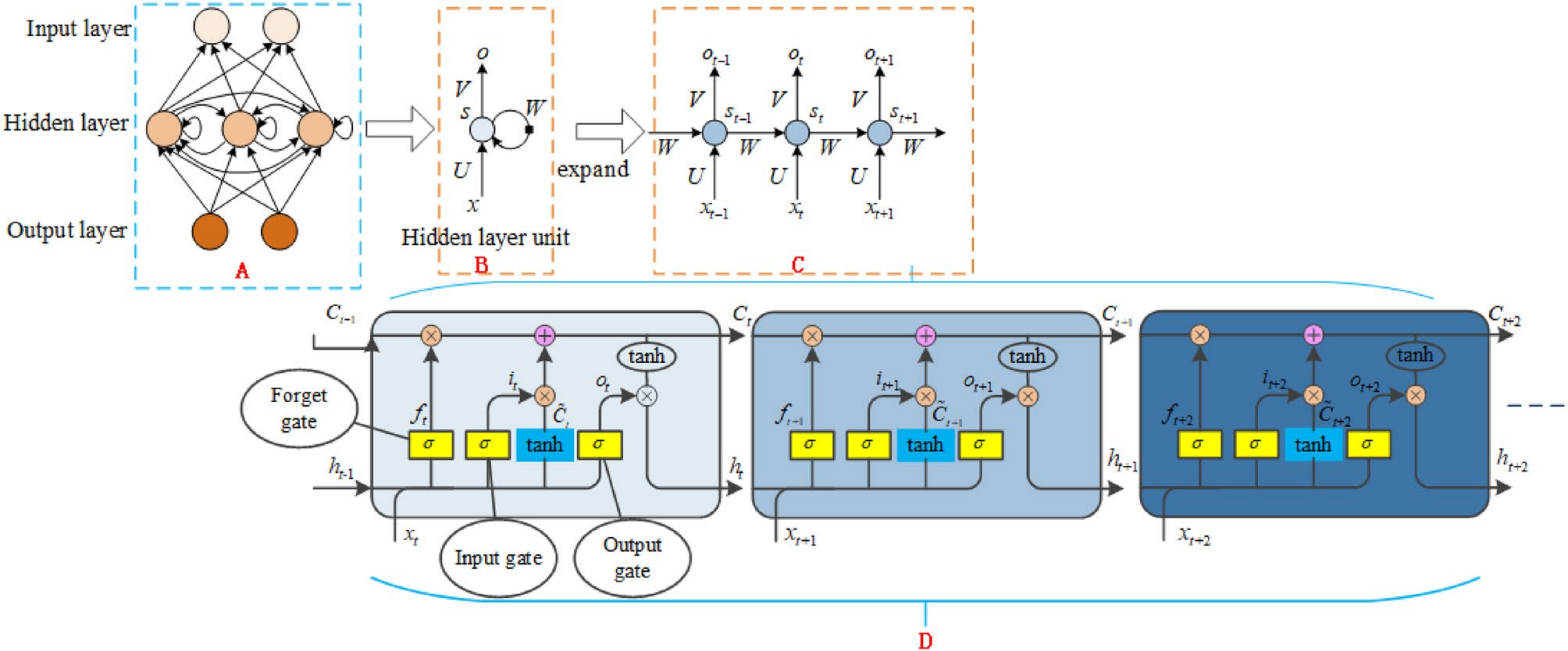
Network structure of the LSTM.

The LSTM network is used to extracts temporal features from epileptic EEG. Conventional cyclic neural networks, such as RNNs, can learn complex time information within an input sequence^27^. Their basic structure is depicted in Fig. 4A–C. The hidden layer of the RNN receives external inputs $x_{t}$ at time step $t$, and also acquires the hidden layer state $S_{t - 1}$ from the previous time step *t*-1. The hidden layer state $S_{t}$ is also saved, and the outputs $o_{t}$ are generated through calculation, where $U$ denotes the weight matrix of the input connection, $V$ represents the weight matrix of the output connection, and $W$ is the weight matrix of the state loop connection.

When the input sequence becomes too long, the network may encounter the vanishing or exploding gradient problem, making it challenging to learn information from longer time contexts. To address this issue, the LSTM’s hidden layer is designed with connections not only to the preceding and succeeding nodes, but also its own nodes, forming a recurrent structure. This allows the LSTM to map the sequence formed by all historical inputs the output sequence, making it theoretically capable of approximating any time series to sequence mapping.

The LSTM network is selected to further extract timing information from the EEG data. The output of the upper layer, specifically the pooling layer, is used as input to the LSTM. The hidden layer unit of the LSTM consists of three filtering gates, namely the, forget gate, input gate, and output gate, corresponding to the three sigmoid layers $\sigma$ shown in Fig. 4D. The symbols $\oplus$ and $\otimes$ represent point-wise addition and point-wise multiplication operations, respectively.

**Figure 5:**
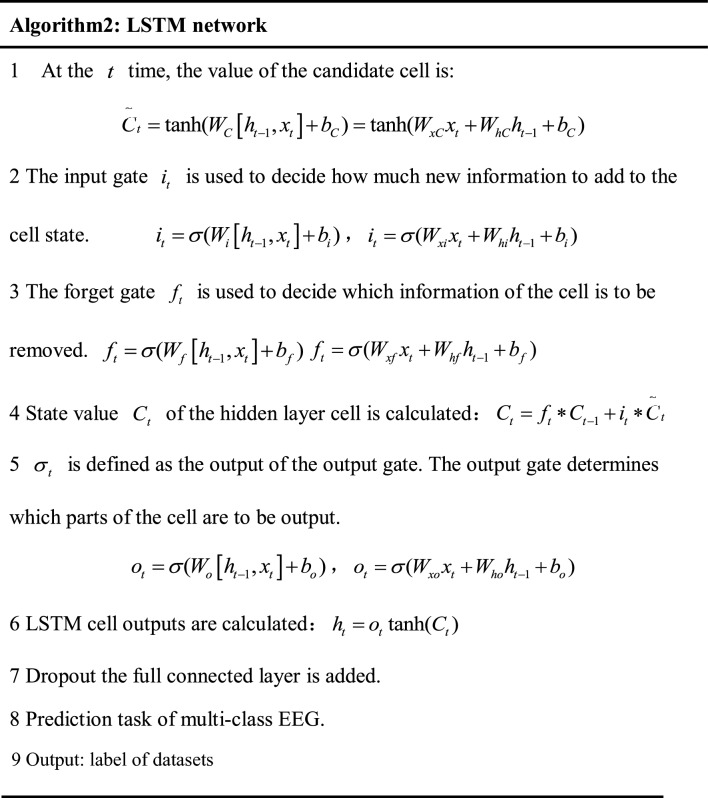
Pseudocode of LSTM algorithm. where $x_{t}$ is the input data at time step $t$,$h_{t - 1}$ is the output data of the hidden layer at time step $t - 1$, $\sigma$ represents a logistic sigmoid function, operator ‘$\cdot$’ indicates matrix multiplication while ‘$*$’ represent element-wise multiplication, $W_{f} ,W_{i} ,W_{o} ,W_{C}$ are the weight matrices, and $b_{f} ,b_{i} ,b_{o} ,b_{C}$ are corresponding offset vector; The vectors $h_{t - 1}$ and $x_{t}$ are combined and applied in subsequent calculations.

The specific calculation steps are presented in Fig. 5.

To prevent over-fitting, the dropout layer is introduced during the forward propagation process. This technique involves randomly deactivating the activation values of certain neurons with a specific probability P. By doing so, the dependency between adjacent neurons is weakened, leading to an improved generalization ability of the network. Subsequently, the softmax function is applied in the fully-connected layer to perform the final classification prediction task for the multi-class EEG.

#### 10-tenfold cross validation

**Figure 6:**
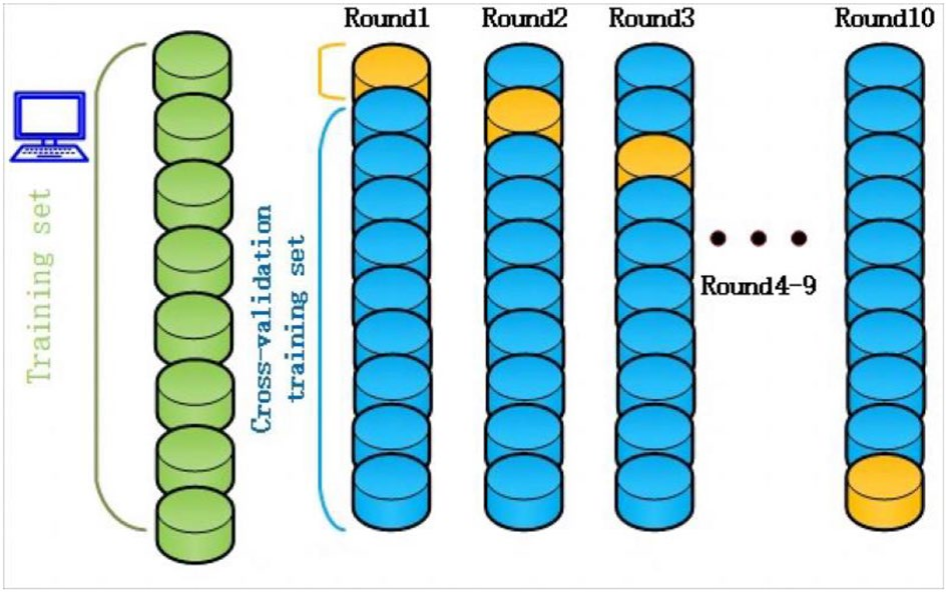
10-tenfold CV training method.

To reduce the influence of the selected training and test data on the model evaluation, tenfold cross validation (CV) was used to split the datasets. This involves the training set being randomly divided into 10 subsets without repetition. 10-1subsets were trained, and the remaining only one subset was test. The average error of the qth test set is calculated as follows. $CVe = \frac{1}{10}\sum\limits_{q = 1}^{10} {e_{q} }$ This process was repeated 10 times to obtain ACCs, which were average to provide a mean value as the final ACC. $e_{q} = \frac{1}{m}\sum\limits_{n = 1}^{m} {\left( {\mathop {y_{n} }\limits^{ \wedge } - y_{n} } \right)^{2} }$ Here, m is the number of samples in the qth test set. $\hat{y}_{n}$ is the predicted value,$y_{n}$ is the real value. This split method can make the classified network more reliable and credible. The 10-CV process is shown in Fig. 6.

## Experimental data

This experiment used an open-source database available at Bonn University, Germany (http://epileptologie-bonn.de/cms/upload/workgroup/lehnertz/eegdata.html)^28^. Datasets have been widely used to test models proposed by many researchers and are considered a benchmark for developing seizure recognition systems. The noninvasive EEG datasets were obtained from 25 subjects with medically intractable partial epilepsy.

The datasets consist of five sub-datasets of ictal scalp EEG: {F, N, O, Z, and S}. Each subset respectively contains 100 data samples from subjects that including experienced epileptologists and healthy individuals. Based on the severity of the disease, this study combines them into three types {F}, {N}-{O}, {Z}-{S}. More detailed information about the dataset can be found in Table 1.

**Table 1:** Dataset description.

Parameter Description	Dataset category	Subject condition	Epileptogenic foci	Electrode collection area	Samples number	Data parameter
5 small sample datasets, 4096 data points	{O}	Healthy subjects	Scalp surface	All brain areas	100	173.61 Hz band-pass filter
	{Z}	Healthy subjects	Scalp surface	All brain areas	100	
	{F}	Intermittent epilepsy	Intracranial	Lesion outside inside area	100	0.53–40 Hz (12 dB/oct)
	{N}	Intermittent epilepsy	Intracranial	Lesion outside inside area	100	
	{S}	Continuous Ictal epilepsy	Intracranial	Intra lesional area	100	Segment duration 23.6s

The main goal of this study is to classify the {F}, {N}–{O}, {Z}–{S} subsets into the three classes. The subsets {O}, {Z} were collected from healthy subjects. The subjects of subsets {F}, {N} were epilepsy patients who suffer an intermittent seizure by the intracranial EEG during the data acquisition period. The subset {S} was obtained from epilepsy patients with continuous epilepsy episodes originating from lesions within the area of the intracranial EEG.

## Results

**Figure 7:**
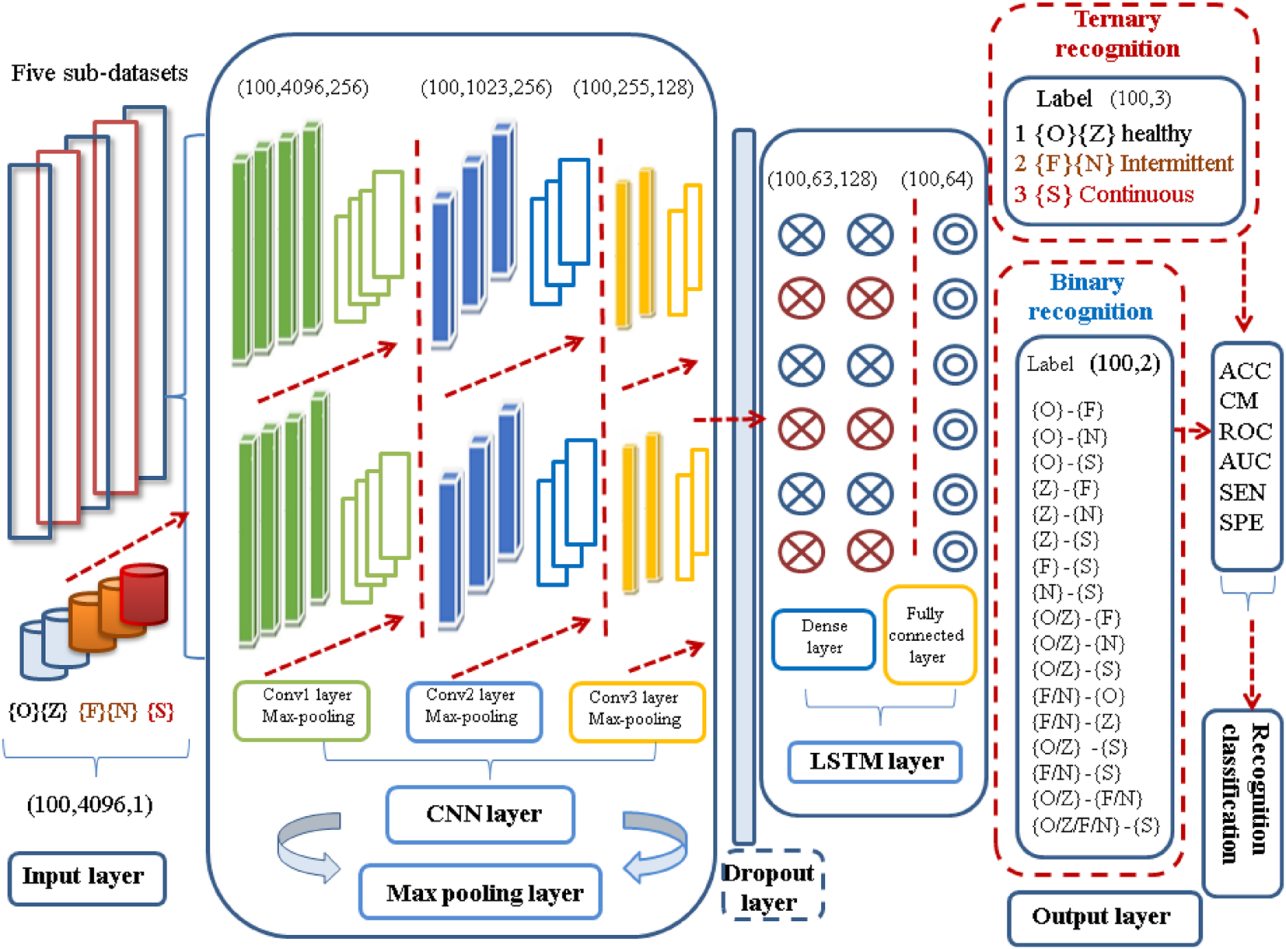
Training flow of the CNN-LSTM model.

Our proposed combined CNN and LSTM method is an integration of CNN and LSTM. First, the CNN part of the model processes the data, and the one-dimensional results are input into the LSTM model to predict the categories {F, N, O, Z, and S}. The joint spatio-temporal CNN-LSTM network automated recognition of epilepsy has several innovations and advantages for multi-class recognition of epileptic EEG. The training frame of the model is illustrated in Fig. 7.

In the application of deep learning indices for the assessment of the diagnosis system, solely pursuing the high classification accuracy of the diagnosis rate cannot provide a comprehensive evaluation of the classification effect. Several other verification indicators, namely ACC, CM, PRC, ROC, and AUC, help to reveal the reasons behind the recognition rates in auxiliary medical diagnosis. Specificity (SPE) and Sensitivity (SEN) of {O/Z}, {F/N}, and {S} are defined as:

$$\frac{{A_{22} + A_{23} + A_{32} + A_{33} }}{{A_{2} + A_{3} }}$$, $$\frac{{A_{11} + A_{13} + A_{31} + A_{33} }}{{A_{1} + A_{3} }}$$, $$\frac{{A_{11} + A_{12} + A_{21} + A_{22} }}{{A_{1} + A_{2} }}$$, $$\frac{{A_{11} }}{{A_{1} }}$$,$$\frac{{A_{22} }}{{A_{2} }}$$,$$\frac{{A_{33} }}{{A_{3} }}$$.

The parameters $$A_{ij}$$$$\left( {i = j} \right)$$ denote the correct classification probability of subset $$\left\{ i \right\}$$ among the five sub-datasets, while $$A_{ij}$$$$\left( {i \ne j} \right)$$ denotes the incorrect probability. The parameters $$A_{i} = \sum\limits_{i = 1}^{3} {A_{1i} }$$ are the sum of all classification rates of each subset $$\left\{ i \right\}$$$$\left( {i,j = 1,2,3} \right)$$ as shown in Table 2.

**Table 2:** Definition of the classification index of sub-datasets.

Test\real type	{O/Z}	{F/N}	{S}
{O/Z}	$A_{11}$	$A_{12}$	$A_{13}$
{F/N}	$A_{21}$	$A_{22}$	$A_{23}$
{S}	$A_{31}$	$A_{32}$	$A_{33}$

This study summarizes the representative results of the epileptic dataset of Bonn University over recent years, including the recognition strategies used, the classification levels, and the processing results of multiple-index evaluations. Previous researchers have classified the data into binary^29,30^ according to Table 3. However, Yuanfa Wang et al. conducted ternary classification based on {F/N}–{O/Z}–{S}, and achieved an ACC rate of 93.6%^31^. Recently, Ilakiyaselvan N et al. used the CNN classifier and obtained an ACC rate of 96%^32^. In contrast, our approach outperformed these methods in the ternary problem, achieving an accuracy of 98% using the hybrid CNN-LSTM model. Based on the above experimental results, we can infer that our proposed deep learning hybrid scheme of CNN and LSTM has the potential for both binary and ternary automated classification of epileptic EEG.

**Table 3:** Comparison of the ACC of the binary and ternary classifications.

Authors	Strategies	Dataset	ACC	AUC	CM/PRC
Samiee et al. (2015)^29^	STFT spectral coefficients with their statistical, values, Bayes, LR, SVM, KNN, ANN	{Z}–{S}	99.8	No	No
		{O}–{S}	99.3		
		{N}–{S}	98.5		
		{F}–{S}	94.9		
		{F/N/O/Z}–{S}	98.1		
Swami et al. (2016)^9^	DTCWT, energy an std, Shannon entropy features, RNN	{Z}–{S}	100	No	No
		{O}–{S}	98.89		
		{N}–{S}	98.72		
		{F}–{S}	93.3		
		{Z/O}–{S}	99.1		
		{N/F}–{S}	95.1		
		{F/N/O/Z}–{S}	95.2		
Sharma et al. (2017)^30^	ATFFWT and FD, LS-SVM	{Z}–{S}	100	No	No
		{O}–{S}	100		
		{N}–{S}	99		
		{F}–{S}	98.5		
		{Z/O}–{S}	100		
		{N/F}–{S}	98.6		
		{Z/O}–{N/F}	92.5		
		{F/N/O/Z}–{S}	99.2		
Yuanfa et al. (2018)^31^	DWT, SVM	{F/N}–{O/Z}–{S}	93.9	No	No
Ilakiyaselvan et al.^32^	RPS, CNN	{Z}–{S}	100	No	Yes
		{O}–{S}	99.4		
		{N}–{S}	98.8		
		{F}–{S}	97.2		
		{Z/O}–{S}	99.2		
		{N/F}–{S}	97.7		
		{Z/O}–{N/F}	98.1		
		{F/N/O/Z}–{S}	98.6		
		{F/N}–{O/Z}–{S}	96		
This work	Hybrid 3 CNN-1 LSTM network model	{Z}–{S}	100	Yes 3-level 96.8	Yes
		{O}–{S}	100		
		{N}–{S}	98.2		
		{F}–{S}	97.6		
		{Z/O}–{S}	98.3		
		{N/F}–{S}	97.9		
		{Z/O}–{NF}	97.3		
		{F/N/O/Z}–{S}	98.7		
		**{F/N}**–**{O/Z}**–**{S}**	**98.0**		

*KNN* k-nearest neighbor, *ANN* artificial neural networks, *SVM* support vector machine, *LS*-*SVM* least square-support vector machine, *RNN* regression neural network, *DWT* discrete wavelet transform, *CNN* convolution neural network.

Significant values are in bold.

**Figure 8:**
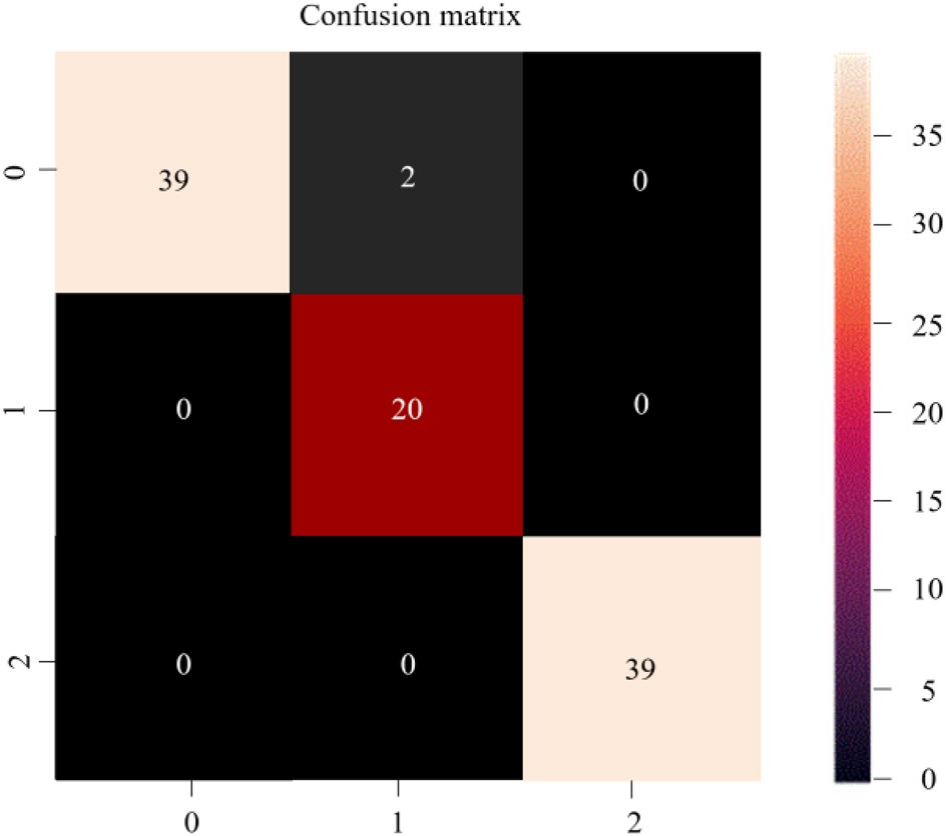
CM evaluation for {F/N}-{O/Z}-{S}.

Furthermore, we compared the CM for the ternary datasets {S}–{F/N}–{O/Z} of ictal epilepsy in Fig. 8, and intermittent epilepsy patients, as well as healthy subjects, using the CNN-LSTM network model. This comparison highlights that the algorithm not only ensures high prediction accuracy for true positives and true negatives, as depicted on the main diagonal line, but also helps to avoid errors related to false positives and true negatives, which is represented by the off-diagonal line.

**Figure 9:**
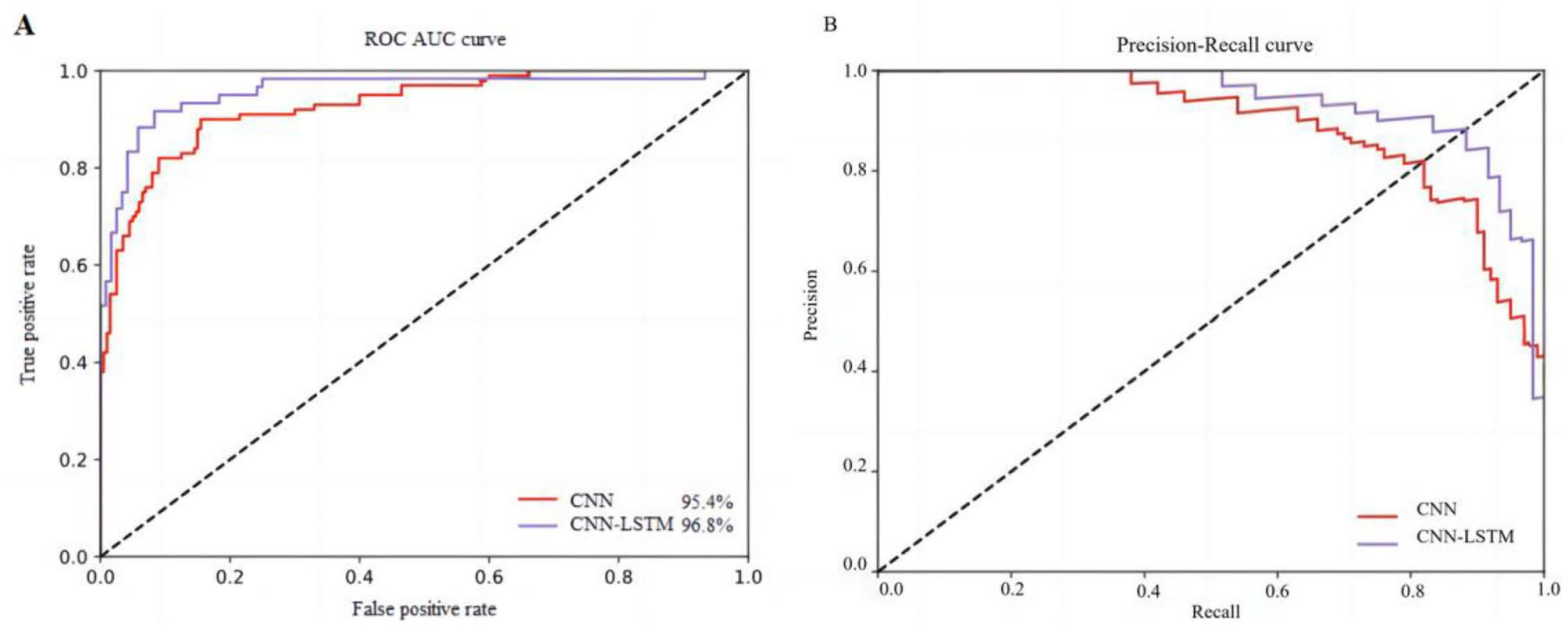
Comparison of the ROC curve and PRC.

With the development of deep learning in recent years, the recognition ACC and CM may not be insufficient to fully assess the classification performance of a model. To ensure a comprehensive evaluation, we added have incorporated the ROC and AUC index. The AUC provides a natural measure for overall assessment of a model’s performance based on the ROC. Li et al. also used the AUC for their analysis results using the same subset, but their values ranged from 0.66 to 0.87, which were not highly effective^23^. In contrast, the AUC values obtained with our proposed CNN-LSTM network model, compared to the CNN model using subsets {F/N}–{O/Z}–{S}, were 95.4% and 96.8%, respectively, as shown in Fig. 9A. In medical diagnostic recognition, it is crucial to achieve a high true-positive rate while maintaining a fixed lower-false positive rate.

The data presented in Fig. 9B reveal that the hybrid CNN-LSTM model outperforms other models in ternary recognition as evidenced by the ACC, CM, ROC curve, AUC, and PRC.

## Discussion

The references in Table 3 use classical machine learning methods to address complex nonlinear data. However, these approaches not only require labeled training and testing data, but also entail complicated parameter adjustments in a supervisory network. EEG are complex nonlinear stochastic timing signals and with high dimension multi-channels, making them difficult to model.

Deep learning, on the other hand, possesses powerful learning capabilities to process nonlinear and high-dimensional data. Deep learning models can automatically learn and extract relevant information from the original data. Through convolution operations, the original signals can be enhanced, the noise is reduced, and signal-to-noise ratio improved. The use of weight sharing strategies can significantly reduce the computational burden. As a result, we aim to apply the deep learning methods for the recognition and analysis of epileptic EEG.

Some researchers detected EEG by using independent machine learning and deep learning model, such as ANN, KNN, and CNN, achieve relatively good result. However, use of multi-channel electrodes corresponds to the spatial information of different regions of the brain. Therefore, some valuable hidden useful information may not be fully utilized during the process, resulting in the lower classification ACC.

In this paper, a hybrid approach of CNN and LSTM is used to extract and classify epileptic EEG. This combination effectively addresses the issue of low classification rate in EEG due to low signal-to-noise ratios. The CNN is component excels in feature extraction and can also locate and predict key points, while the LSTM component more effectively explores the timing information within the EEG. The hybrid CNN-LSTM model compensates for the compensates of using only CNN.

The LSTM network is improved by introducing a gate structure in each LSTM unit. This gate structure allows each element output by the sigmoid layer to be a real number between 0 and 1, revealing the weight or importance of the corresponding information. It determines how information from the previous inputs is forgotten and updates as new inputs become available in the long range sequence. Eventually, the time series information of the extracted EEG data passes through the dropout and the fully-connected layer to complete the classification task. Thus, hybrid CNN-LSTM method maximizes the temporal and spatial information of epileptic EEG, leading to improve recognition ACC.

This method is more convenient and faster than the classical machine learning model, and it alleviates concerns about the changes in model training with accumulating medical data increases. Simultaneously, hybrid CNN-LSTM model not only addresses the binary recognition issues, but also tackles ternary and more complex classification challenges with better extension capabilities.

## Conclusions

The use of EEG has revolutionized the recognition epileptic seizures. In this study, we proposed a dual stream spatiotemporal hybrid network, that is, combined CNN–LSTM algorithm for an auxiliary medical diagnostic system for Epileptic EEG, which demonstrated excellent performance in a ternary classification of healthy subjects, intermittent epilepsy patients, and continuous ictal epilepsy patients. The pre-processed data are input to the CNN comprising three convolution layers and three pooling layers, extract spatial features of the epileptic EEG data are extracted by convolution pooling. The spatial characteristics of the EEG data are then directly input into the LSTM network, extracting time domain information from the EEG data. Unlike other approaches that have mainly focused on accuracy rates, we prioritized the use of additional indices to evaluate the potential for misclassification using DL technologies. These indicators are crucial for medical screening. The experimental results conclusively demonstrated the effectiveness of our proposed hybrid CNN-LSTM approach in achieving favorable result in binary and ternary classifications. The method even can be better extended to solve multiple classification problems. Additionally, the method avoids manual operation and is more beneficial for doctors to diagnose during the clinical process to ease the workload of the neurologist.

As part of our future work, we plan to explore this scheme for detecting EEG signals to complementary medicine will go out of the laboratory. This method can replace of doctor-patient interactions with an effective DL algorithm and develop an epilepsy detection mobile application. It is possible to integrate some intelligent sleep analysis into the software. EEG signals can be transmitted to mobile terminals through wireless sensors, which used for detecting and alerting at night. The technology can prevent sudden death of epilepsy patients or infants at night. It will improve the quality of life for patients and caregivers.

## Data Availability

Please refer to the data source provided by University of Bonn. EEG time series download page. http://epileptologie-bonn.de/cms/upload/workgroup/lehnertz/eegdata.html.
